# Geranyl Acetate Attenuates Para-phenylenediamine-induced Cytotoxicity, DNA Damage, Apoptosis, and Inflammation in HaCaT Keratinocytes

**DOI:** 10.5812/ijpr-164379

**Published:** 2025-10-01

**Authors:** Jae Ho Lee, Sungkwan An, Seokmuk Park, Seunghee Bae

**Affiliations:** 1 Department of Biological Engineering, Konkuk University, Seoul, Republic of Korea; 2 Dermato Bio, Inc., Gyeonggi-do, Republic of Korea

**Keywords:** Para-phenylenediamine, HaCaT Keratinocytes, Geranyl Acetate, Plant Metabolites, Dermatitis

## Abstract

**Background:**

As consumer demand for cosmetic products that enhance physical appearance continues to rise, the global oxidative hair dye market is experiencing steady growth. Para-phenylenediamine (PPD), a key ingredient in most oxidative hair dyes, is widely used due to its efficacy and low cost. However, its high chemical reactivity has been consistently linked to adverse effects, including allergic contact dermatitis (ACD), eczema, carcinogenicity, and genotoxicity.

**Objectives:**

Given the concerns over the long-term use of conventional therapies such as topical corticosteroids (TCS) and calcineurin inhibitors, this study aimed to identify a plant-derived compound with protective properties in a keratinocyte model of PPD-induced toxicity.

**Methods:**

To assess the cytoprotective potential of 14 selected plant metabolites, water-soluble tetrazolium salt-1 (WST-1) and lactate dehydrogenase (LDH) assays were performed. Western blotting and reverse transcription-polymerase chain reaction were used to evaluate the anti-apoptotic, anti-DNA damage, and anti-inflammatory effects of geranyl acetate (GA), the most promising candidate.

**Results:**

Among the 14 tested plant metabolites, GA was identified as the most effective compound in mitigating cytotoxicity in HaCaT keratinocytes. Co-treatment with GA significantly attenuated PPD-induced phosphorylation of p53 and MAPK, indicating inhibition of the DNA damage response (DDR) pathway. Further experiments revealed that GA suppressed the upregulation of apoptosis-related proteins [p53 upregulated modulator of apoptosis (PUMA), B-cell lymphoma 2 (BCL-2)-associated X protein (BAX), cytochrome c, and cleaved poly (ADP-ribose) polymerase (PARP)]. Moreover, GA treatment decreased the phosphorylation of signal transducer and activator of transcription 3 (STAT3) and NF-κB p65, thereby downregulating five pro-inflammatory cytokines [interleukin (IL)-1α, IL-1β, IL-6, tumor necrosis factor (TNF)-α, and IL-24] and five chemokines [C-C motif chemokine ligand (CCL) 5/RANTES, CCL20/MIP-3α, CCL26/eotaxin-3, C-X-C motif chemokine ligand (CXCL) 1/GRO-α, and CXCL8/IL-8], confirming its anti-inflammatory efficacy.

**Conclusions:**

Collectively, this study suggests GA as a promising plant-derived metabolite with cytoprotective, genoprotective, anti-apoptotic, and anti-inflammatory effects in PPD-stimulated HaCaT cells.

## 1. Background

Contact dermatitis (CD) is an inflammatory skin disorder that arises from exposure to irritant substances or allergenic agents. It is clinically characterized by edema, erythema, oozing, a burning sensation, and pruritus. Contact dermatitis is broadly categorized into two main types: Allergic contact dermatitis (ACD) and irritant contact dermatitis (ICD) (1). Among these, ICD accounts for approximately 80% of cases, while ACD represents about 20% (2). In contrast to ICD, which can often be managed by avoiding physical or chemical irritants, ACD presents greater therapeutic challenges due to the difficulty in accurately identifying and avoiding specific allergens (3). Current treatment strategies for ACD include topical corticosteroids (TCS), topical calcineurin inhibitors (TCIs), and phototherapy (PT) (2, 4). However, the use of TCS is frequently associated with adverse effects, including skin atrophy, fungal infections, and acne, and prolonged use may lead to systemic side effects such as adrenal suppression and osteoporosis, thus necessitating careful and limited application (4-6). The TCIs have been linked to pruritus, erythema, and potential carcinogenicity, while PT has been associated with actinic keratosis, solar lentigines, and an increased risk of skin cancer (4, 7, 8). Moreover, ACD affects up to 20.1% of the general population and as much as 48.2% of occupational contact dermatitis cases, imposing a substantial socioeconomic burden due to lost productivity, increased healthcare costs, and disability-related compensation (9-13). These challenges underscore the pressing need to discover and develop new therapeutic agents for the prevention and management of ACD.

To date, more than 3,000 substances have been identified as potential ACD-inducing allergens. Among these, para-phenylenediamine (PPD) is recognized as a major sensitizer, alongside nickel, cobalt, Myroxylon pereirae (balsam of Peru), and chromium (2, 8). The PPD is an aromatic amine that has long been used as a key component in oxidative hair dyes since the late 19th century and is estimated to account for up to 6% of ACD cases (14). Because of its low production cost, PPD is found in approximately 80% of hair dye formulations. However, skin exposure to PPD has been associated not only with ACD but also with carcinogenicity, acute dermatitis, systemic toxicity, and disruption of skin barrier integrity, warranting extreme caution in its use (15-19).

Plants and their metabolites are valuable resources in drug discovery and are extensively used in pharmaceuticals, foods, cosmetics, and other industries (20). They produce a vast array of organic compounds, broadly classified as primary metabolites, essential for growth and development, and secondary metabolites, which protect against environmental stressors (20, 21). These metabolites can be grouped by chemical structure, solubility, or biosynthetic pathway into six major classes — polyphenols, terpenoids, carotenoids, alkaloids, glucosinolates, and sulfoxides (22). Limitations in the metabolism and safety of some synthetic drugs have driven interest in plant-derived compounds as safer therapeutic alternatives (23-25).

Plant metabolites exhibit diverse pharmacological properties, including antioxidant, anti-inflammatory, anticancer, antimicrobial, antiallergic, anti-aging, and neuroprotective effects (26-28). Given that PPD is a known inducer of ACD, we screened 14 compounds with reported health-promoting properties to identify the most effective in mitigating its effects in HaCaT cells (29-36). To capture the structural diversity of plant metabolites, candidates were selected to represent the six major classes — polyphenols, terpenoids, carotenoids, alkaloids, glucosinolates, and sulfoxides.

Among the tested compounds, geranyl acetate (GA) most effectively reduced PPD-induced cytotoxicity and inflammation in HaCaT cells. This terpenoid, present in carrots and other plants, has been shown to suppress nitrite production in RAW 264.7 cells, exhibit antinociceptive effects in mice, and display antimicrobial, antifungal, and anticancer activities (33, 37-40). Widely used in food and cosmetic products, it is non-genotoxic, non-photoirritant, and non-photoallergenic, with the No Expected Sensitization Induction Level (NESIL) of 5 mg/cm², indicating a low risk of skin sensitization even at high concentrations (41). These attributes support its potential as a safe, plant-derived metabolite for mitigating PPD-induced inflammation and cytotoxicity in keratinocytes.

## 2. Objectives

In this study, we screened 14 plant metabolites with reported health-promoting properties and identified GA as a compound that mitigates PPD-induced cytotoxicity in HaCaT cells. We further investigated its cytoprotective, genoprotective, anti-apoptotic, and anti-inflammatory effects in a PPD-stimulated keratinocyte model. Based on our findings, we propose that GA has pharmacological potential as a therapeutic agent for PPD-related skin disorders.

## 3. Methods

### 3.1. Chemicals and Cell Culture

The PPD (#P6001), p-coumaric acid (#9008), apigenin (#SMB00702), resveratrol (#R5010), limonene (#62118), folic acid (FA, #F7876), lutein (#LRAB3708), berberine (#14050), and salicin (#S0625) were purchased from Sigma-Aldrich (USA). Isoferulic acid (#HY-N0761), regaloside A (#HY-N7931), and alliin (#HY-126085) were obtained from MedChemExpress (USA), while phloroglucinol (PG, #P0249), naringenin (#N0072), and chlorogenic acid (#C1081) were purchased from Tokyo Chemical Industry Co. (Japan). HaCaT human keratinocytes were obtained from Cytion (#300493; Germany) and cultured in DMEM (#LB001-05; Welgene, Republic of Korea) supplemented with 10% (v/v) fetal bovine serum (35-015-CV; Corning, USA). Cells were maintained in a humidified 5% CO_2_ incubator and subcultured at 80% confluence.

### 3.2. Water-Soluble Tetrazolium Salt-1 Assay

HaCaT cells were seeded in 96-well plates (3 × 10^3^ cells/well) and cultured for 24 hours. Before evaluating the cytoprotective effects of the 14 plant metabolites on PPD-exposed HaCaT cells, we first determined the appropriate non-cytotoxic concentrations for each compound. Specifically, cytotoxicity was assessed within the following concentration ranges: Naringenin (0 - 100 μM), p-coumaric acid (0 - 4 mM), apigenin (0 - 5 μM), resveratrol (0 - 5 μM), chlorogenic acid (0 - 1 mM), PG (0 - 40 μM), lutein (0 - 20 μM), GA (0 - 1 mM), limonene (0 - 400 μM), FA (0 - 40 μM), salicin (0 - 1 mM), isoferulic acid (0 - 1 mM), alliin (0 - 200 μM), and regaloside A (0 - 200 μM). As shown in Appendix 1, these experiments identified the optimal non-cytotoxic concentrations, which were used in subsequent assays.

For the screening experiments, cells were pretreated with each of the 14 plant metabolites or vehicle for 30 minutes, followed by co-treatment with PPD for up to 72 hours. After incubation, the cells were washed once with DPBS (#LB001-02; Welgene), and 100 μL of EZ-Cytox reagent (#EZ-500; DoGenBio, Republic of Korea) was added to each well. After a 30-minute incubation at 37°C, absorbance was recorded at 450 nm using a microplate reader.

### 3.3. Lactate Dehydrogenase Leakage Assay

HaCaT cells were plated in 96-well plates at a density of 3 × 10³ cells per well and pretreated for 30 minutes with either one of five selected plant-derived metabolites or a vehicle. Subsequently, the cells were exposed to PPD (250 μM) and incubated for 48 hours. Following incubation, 10 μL of the culture supernatant was transferred to a fresh 96-well plate, and 100 μL of EZ-LDH reagent (#DG-LDH500; DoGenBio) was added. After a 30-minute reaction period, absorbance was measured at 450 nm.

### 3.4. Western Blot Analysis

HaCaT cells were plated in 100 mm culture dishes at a density of 2 × 10^5^ cells per dish and incubated for 24 hours. The cells were pretreated with plant metabolites or vehicle for 30 minutes, followed by co-treatment with PPD (250 μM) for 48 hours. After treatment, the cells were lysed for 30 minutes in RIPA buffer. Western blotting was carried out as previously described (32), and protein bands were detected using the Pierce^™^ ECL Substrate (#32106; Thermo Fisher Scientific). Information on the primary antibodies utilized is summarized in Table 1. 

**Table 1.** List of Primary Antibodies for Western Blot Analyses

Antigen	Host	Dilution	Manufacturer (Cat. Number)
**β-Actin**	Mouse	1:1000	Santa Cruz (#sc-47778)
**Cleaved Caspase-3**	Rabbit	1:1000	CST (#9664)
**Cleaved PARP**	Rabbit	1:1000	CST (#5625)
**BCL-2**	Rabbit	1:1000	Abcam (#59348)
**BAX**	Rabbit	1:1000	CST (#5023)
**p65**	Rabbit	1:1000	CST (#8242)
**p-p65 (Ser468)**	Rabbit	1:1000	CST (#3039)
**p-ATR (Ser428)**	Rabbit	1:1000	CST (#2853)
**ATR**	Rabbit	1:1000	CST (#13934)
**p-p53 (Ser9)**	Rabbit	1:1000	CST (#9288)
**p-p53 (Ser15)**	Rabbit	1:1000	CST (#9284)
**p-p53 (Ser46)**	Rabbit	1:1000	CST (#2521)
**p-p53 (Ser392)**	Rabbit	1:1000	CST (#9281)
**p53**	Mouse	1:200	Santa Cruz (#sc-126)
**p-p38 (Thr180/Tyr182)**	Rabbit	1:1000	CST (#9211)
**p38**	Rabbit	1:1000	CST (#54470)
**p-JNK (Thr183/Tyr185)**	Rabbit	1:1000	CST (#9251)
**JNK**	Rabbit	1:1000	CST (#9252)
**p-ERK (Thr202/Tyr204)**	Rabbit	1:1000	CST (#9101)
**ERK**	Rabbit	1:1000	CST (#9102)
**PUMA**	Rabbit	1:1000	Abcam (#9643)
**Cytochrome c**	Mouse	1:1000	BD Biosciences (#556432)
**p-STAT3 (Ser727)**	Rabbit	1:1000	CST (#94994)
**STAT3**	Rabbit	1:1000	CST (#4904)
**IκB-α**	Rabbit	1:1000	CST (#9242)
**p-IκB-α (Ser32)**	Rabbit	1:1000	CST (#2859)

Abbreviations: PARP, poly (ADP-ribose) polymerase; BCL-2, B-cell lymphoma 2; BAX, B-cell lymphoma 2-associated X protein; ATR, ataxia telangiectasia and Rad3 related protein; JNK, c-Jun N-terminal kinases; ERK, extracellular signal-regulated kinases; PUMA, p53 upregulated modulator of apoptosis; STAT3, signal transducer and activator of transcription 3; IκB-α, NF-kappa-B inhibitor alpha.

### 3.5. Reverse Transcriptase-Polymerase Chain Reaction

HaCaT cells were plated in 100 mm culture dishes at a density of 2 × 10^5^ cells per dish and incubated for 24 hours. The cells were pretreated with GA (0 - 500 μM) for 30 minutes, followed by co-treatment with PPD (250 μM) for 48 hours. Total RNA was isolated using RiboEx^™^ reagent (#301-001; GeneAll Biotechnology, Republic of Korea). Subsequently, 1 μg of RNA was used for cDNA synthesis with oligo(dT) primers, 0.1 M DTT, 2.5 mM dNTPs, 5 × First-Strand Buffer, and M-MLV reverse transcriptase (#28025021; Thermo Fisher Scientific, USA). PCR was then performed using the synthesized cDNA, 1.5 mM dNTPs, appropriate primers, reaction buffer, and Taq polymerase. PCR products were visualized using a UV transilluminator (FUSION SOLO S; France). The primer sequences employed for reverse transcriptase-polymerase chain reaction (RT-PCR) analysis are provided in Table 2. 

**Table 2.** Primer Sequences Utilized in Reverse Transcriptase-Polymerase Chain Reaction Analysis

Target mRNA	Primer Sequences	Product Size (bp)
**β-Actin**	F: 5′-CATCGTCCACCGCAAATGCTTC-3′	240
	R: 5′-TCCTCGGCCACATTGTGAACTT-3′	
**IL-1α**	F: 5′-GAGGCCATCGCCAATGACTCAG-3′	183
	R: 5′-ATGTAATGCAGCAGCCGTGAGG-3′	
**IL-1β**	F: 5′-TTCCCTGCCCACAGACCTTCC-3′	116
	R: 5′-TGCATCGTGCACATAAGCCTCG-3′	
**IL-6**	F: 5′-GTAGCCGCCCCACACAGA-3′	101
	R: 5′-CATGTCTCCTTTCTCAGGGCTG-3′	
**TNF-α**	F: 5′-CTCTTCTGCCTGCTGCACTTTG-3′	135
	R: 5′-ATGGGCTACAGGCTTGTCACTC-3′	
**IL-24**	F: 5’-GGACGTAGAAGCAGCTCTGACCA-3’	184
	R: 5’-AAGGGCGTGAAGTGTCCAGTGA-3’	
**CCL5/RANTES**	F: 5’-CCTGCTGCTTTGCCTACATTGC-3’	125, 207
	R: 5’-ACACACTTGGCGGTTCTTTCGG-3’	
**CCL20/MIP-3**	F: 5’-CCAAGAGTTTGCTCCTGGCT-3’	75
	R: 5’-TGCTTGCTGCTTCTGATTCG-3’	
**CCL26/eotaxin-3**	F: 5’-CCAATACAGCCACAAGCCCCTT-3’	263
	R: 5’-CAGAAAAGATTCCGCAGGCTCCC-3’	
**CXCL1/GRO-α**	F: 5’-AGGCCACCTGGATTGTGCCTAA-3’	281
	R: 5’-GCATGTTGCAGGCTCCTCAGAA-3’	
**CXCL8/IL-8**	F: 5’-TCTCTTGGCAGCCTTCCTGA-3’	172
	R: 5’-TTCTGTGTTGGCGCAGTGTG-3’	

Abbreviations: IL, interleukin; TNF, tumor necrosis factor; CCL, C-C motif chemokine ligand; CXCL, C-X-C motif chemokine ligand.

### 3.6. Statistical Analysis

Statistical analyses were performed using triplicate data. Differences among treatment groups were evaluated by one-way analysis of variance (ANOVA) with GraphPad Prism software (version 8.0.1; San Diego, USA). When significant differences were detected, Tukey’s post-hoc test was performed for multiple comparisons. Results are expressed as the mean ± standard deviation (SD), with statistical significance indicated as * P < 0.05, ** P < 0.01, and *** P < 0.001.

## 4. Results

### 4.1. Establishment of a Para-phenylenediamine-induced Toxicity Model in HaCaT Cells

**Figure 1.**
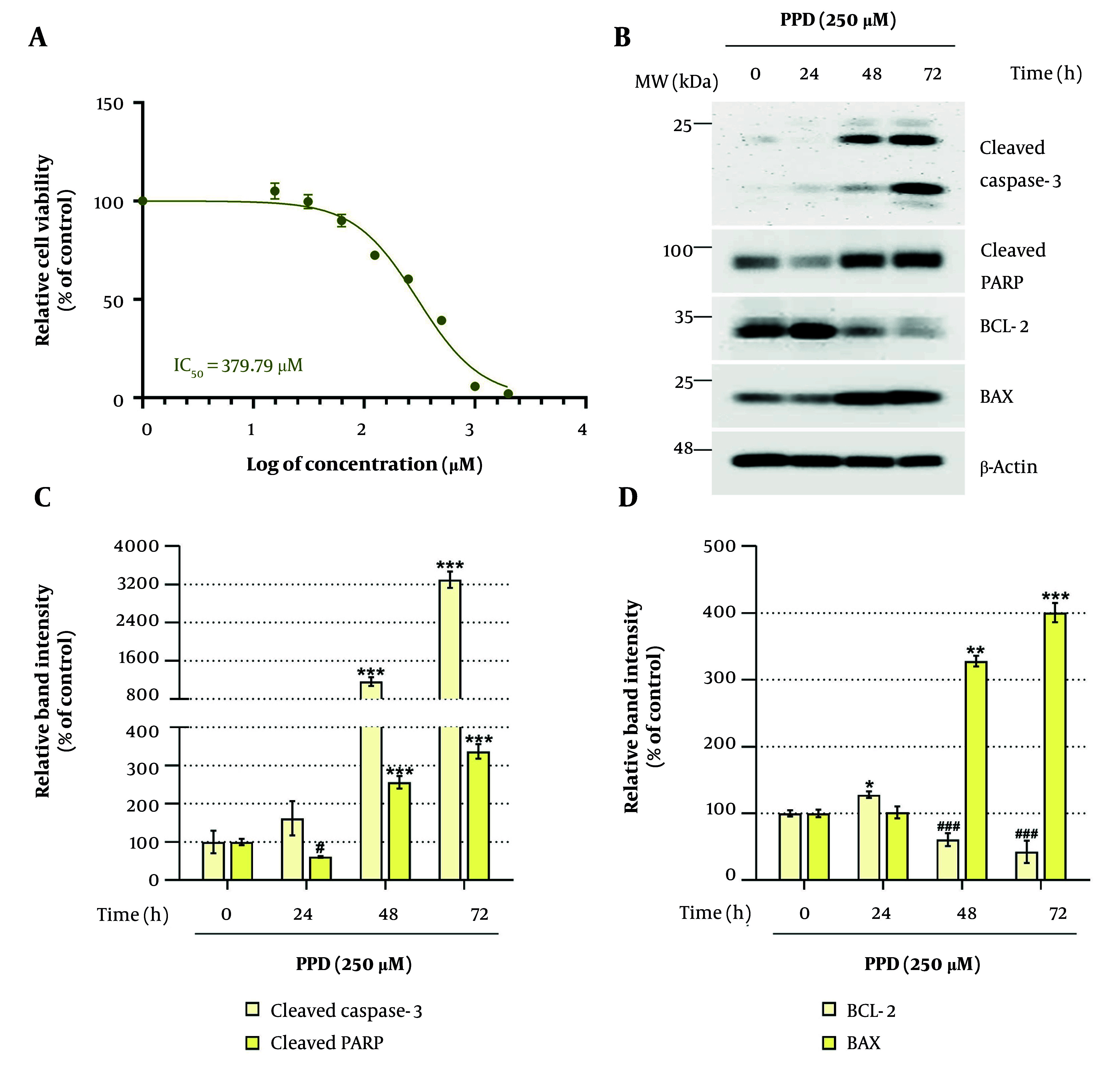
Determination of para-phenylenediamine (PPD)-induced cytotoxicity and apoptosis in HaCaT cells: A, HaCaT cells were seeded in 96-well plates (3 × 10^3^ cells/well) and incubated for 24 hours. The cells were treated with various concentrations of PPD (0 - 1,000 μM) for 48 hours. Cell viability was assessed using the water-soluble tetrazolium salt-1 (WST-1) assay. B – D, Cells were seeded in 100 mm dishes (2 × 10^5^ cells/dish) and incubated for 24 hours. The cells were treated with 250 μM PPD for up to 72 hours. Expression levels of apoptosis-related proteins, including cleaved Caspase-3, cleaved poly (ADP-ribose) polymerase (PARP), B-cell lymphoma 2 (BCL-2), and B-cell lymphoma 2-associated X protein (BAX), were analyzed by Western blotting. The β-Actin was used as a loading control, and protein band intensities were quantified using ImageJ software (version 1.53t). Data are presented as mean ± standard deviation (SD, n = 3). Statistical significance was determined by one-way analysis of variance (ANOVA) followed by Tukey’s post-hoc test (abbreviation: LDH, lactate dehydrogenase. #,* P < 0.05, ** P < 0.01, and ###,*** P < 0.001 compared with the solvent-treated vehicle control group).

To establish a PPD-induced toxicity model in HaCaT cells, we first determined the optimal concentration of PPD that induces approximately 60% cytotoxicity, providing sufficient cellular stress to evaluate toxicity and cell death. As shown in Figure 1A, HaCaT cells were treated with PPD (0 - 1,000 μM) for 48 hours, resulting in an IC_50_ of 379.79 μM. Treatment with 250 μM PPD resulted in a cell viability of approximately 60.25 ± 1.63%. Based on this, we assessed whether 250 μM PPD could upregulate key markers of cell death and apoptosis. As shown in Figure 1B - D, PPD (250 μM) treatment led to a time-dependent increase in cleaved Caspase-3, cleaved poly (ADP-ribose) polymerase (PARP), and B-cell lymphoma 2-associated X protein (BAX) expression, while B-cell lymphoma 2 (BCL-2) expression, an anti-apoptotic marker, was decreased. These results established 250 μM as the working concentration for subsequent experiments aimed at identifying plant-derived compounds with cytoprotective, genoprotective, anti-apoptotic, and anti-inflammatory properties in HaCaT cells.

### 4.2. Geranyl Acetate Confers Cytoprotective Effects Against Para-phenylenediamine-induced Toxicity in HaCaT Cells

**Figure 2.**
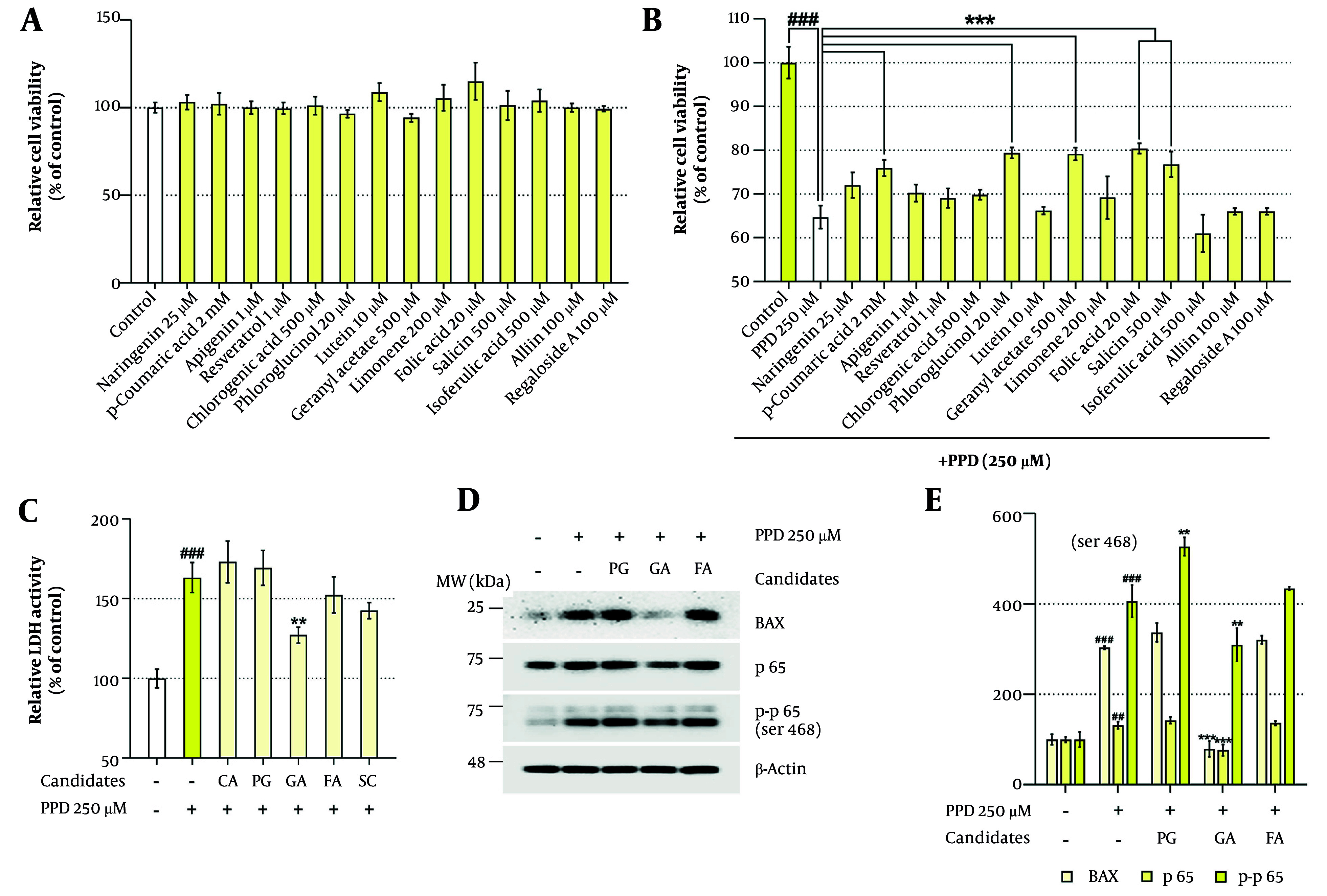
Screening of 14 plant metabolites for mitigating para-phenylenediamine (PPD)-induced cytotoxicity in HaCaT cells: A, HaCaT cells were seeded in 96-well plates (3 × 10^3^ cells/well) and incubated for 24 hours. The cells were treated with indicated concentrations of phytochemical candidates for 48 hours. Cell viability was assessed using the water-soluble tetrazolium salt-1 (WST-1) assay. B and C, To evaluate the protective effects of the plant metabolites against PPD-induced cytotoxicity, HaCaT cells were treated with various concentrations of candidate compounds, in the presence or absence of PPD (250 μM) for 48 hours. Cytotoxicity was assessed by (B) the WST-1 assay and (C) the lactate dehydrogenase (LDH) release assay. D and E, Cells were seeded in 100 mm dishes (2 × 10^5^ cells/dish) and incubated for 24 hours, followed by treatment with phloroglucinol (PG, 20 μM), geranyl acetate (GA, 500 μM), or folic acid (FA, 20 μM), in the presence or absence of PPD (250 μM) for 48 hours. Protein expression levels of the pro-apoptotic marker B-cell lymphoma 2-associated X protein (BAX) and inflammation-associated proteins p65 and p-p65 were analyzed by Western blotting. The β-Actin was used as a loading control. Protein band intensities were quantified using ImageJ software (version 1.53t). Data are presented as mean ± standard deviation (SD, n = 3). Statistical significance was determined by one-way analysis of variance (ANOVA) followed by Tukey’s post-hoc test (## P < 0.01 and ### P < 0.001 compared with the solvent-treated vehicle control group. ** P < 0.01 and *** P < 0.001 compared with the PPD-treated negative control group).

Subsequently, we screened 14 plant metabolites previously reported to have health-promoting properties — naringenin, p-coumaric acid, apigenin, resveratrol, chlorogenic acid, PG, lutein, GA, limonene, FA, salicin, isoferulic acid, alliin, and regaloside A — to identify candidates with protective effects against PPD-induced toxicity in HaCaT cells (29-36). As shown in Appendix 1 in Supplementary File and Figure 2A, non-toxic concentrations of each compound were first determined, followed by co-treatment with PPD (250 μM). Among the 14 metabolites, five — p-coumaric acid, PG, GA, FA, and salicin — significantly improved cell viability compared to the PPD-treated group (Figure 2B). 

To validate these findings, we conducted lactate dehydrogenase **(**LDH) release and western blot assays. Notably, only GA significantly reduced LDH activity from 163.39 ± 9.46% in the PPD-treated group to 127.32 ± 5.01% (Figure 2C). In addition, western blot analysis demonstrated that GA co-treatment attenuated PPD-induced upregulation of BAX (a pro-apoptotic marker) and phosphorylated p65 (a pro-inflammatory marker, Figure 2D and E). Collectively, these results suggest that GA exhibits the most potent cytoprotective activity among the 14 plant metabolites tested.

### 4.3. Geranyl Acetate Attenuates DNA Damage Response in Para-phenylenediamine-treated HaCaT Cells

**Figure 3.**
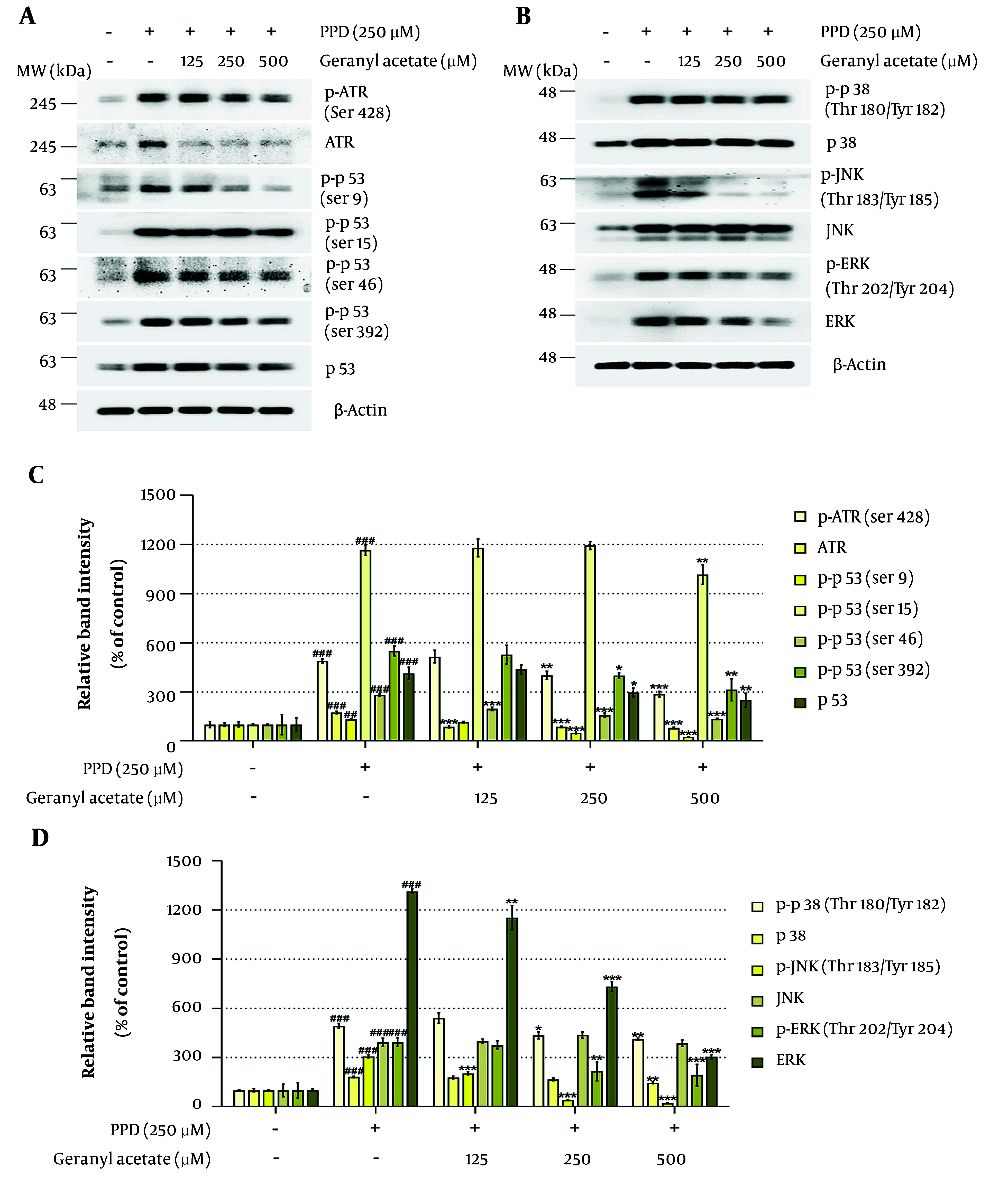
Geranyl acetate (GA) mitigates para-phenylenediamine (PPD)-induced DNA damage response (DDR) signaling in HaCaT cells: A – D, HaCaT cells were seeded in 100 mm dishes (2 × 10^5^ cells/dish) and incubated for 24 hours, followed by treatment with GA (0 - 500 μM), in the presence or absence of PPD (250 μM) for 48 hours. Protein expression levels of the DDR-related proteins, including ataxia telangiectasia and Rad3 related protein (ATR), p-ATR, p53, p-p53, p38, p-p38, c-Jun N-terminal kinases (JNK), p-JNK, extracellular signal-regulated kinases (ERK), and p-ERK, were analyzed by Western blotting. The β-Actin was used as a loading control. Protein band intensities were quantified using ImageJ software (version 1.53t). Data are presented as mean ± standard deviation (SD, n = 3). Statistical significance was determined by one-way analysis of variance (ANOVA) followed by Tukey’s post-hoc test (## P < 0.01 and ### P < 0.001 compared with the solvent-treated vehicle control group. * P < 0.05, ** P < 0.01, and *** P < 0.001 compared with the PPD-treated negative control group).

The PPD is a highly reactive aromatic amine that readily oxidizes into benzoquinone diimine (BQDI) and Bandrowski’s base (BB). Due to its high reactivity, PPD can induce DNA fragmentation, form hapten-protein adducts via nucleophilic interactions, and generate reactive oxygen species (42, 43). It also exhibits strong mutagenic potential through DNA adduct formation and strand breaks, thereby contributing to genotoxicity (43-45). We therefore investigated whether GA could alleviate PPD-induced DNA damage. As shown in Figure 3A and C, co-treatment with GA led to a dose-dependent decrease in ataxia telangiectasia and Rad3 related protein (ATR) phosphorylation, an early marker of DNA damage response (DDR) activation. The ATR signaling is known to drive the downstream phosphorylation of p53 and MAPKs, contributing to inflammation, senescence, and apoptosis (46, 47). Consistently, GA co-treatment also reduced PPD-induced phosphorylation of p53 (Ser9, Ser15, Ser46, and Ser392) and MAPKs [p-p38, p-c-Jun N-terminal kinases (JNK), and p-extracellular signal-regulated kinases (ERK)], as shown in Figure 3A - D. These findings suggest that GA mitigates DNA damage and downregulates DDR signaling pathways in PPD-exposed HaCaT cells.

### 4.4. Geranyl Acetate Exerts Anti-apoptotic Effects in Para-phenylenediamine-treated HaCaT Cells

**Figure 4.**
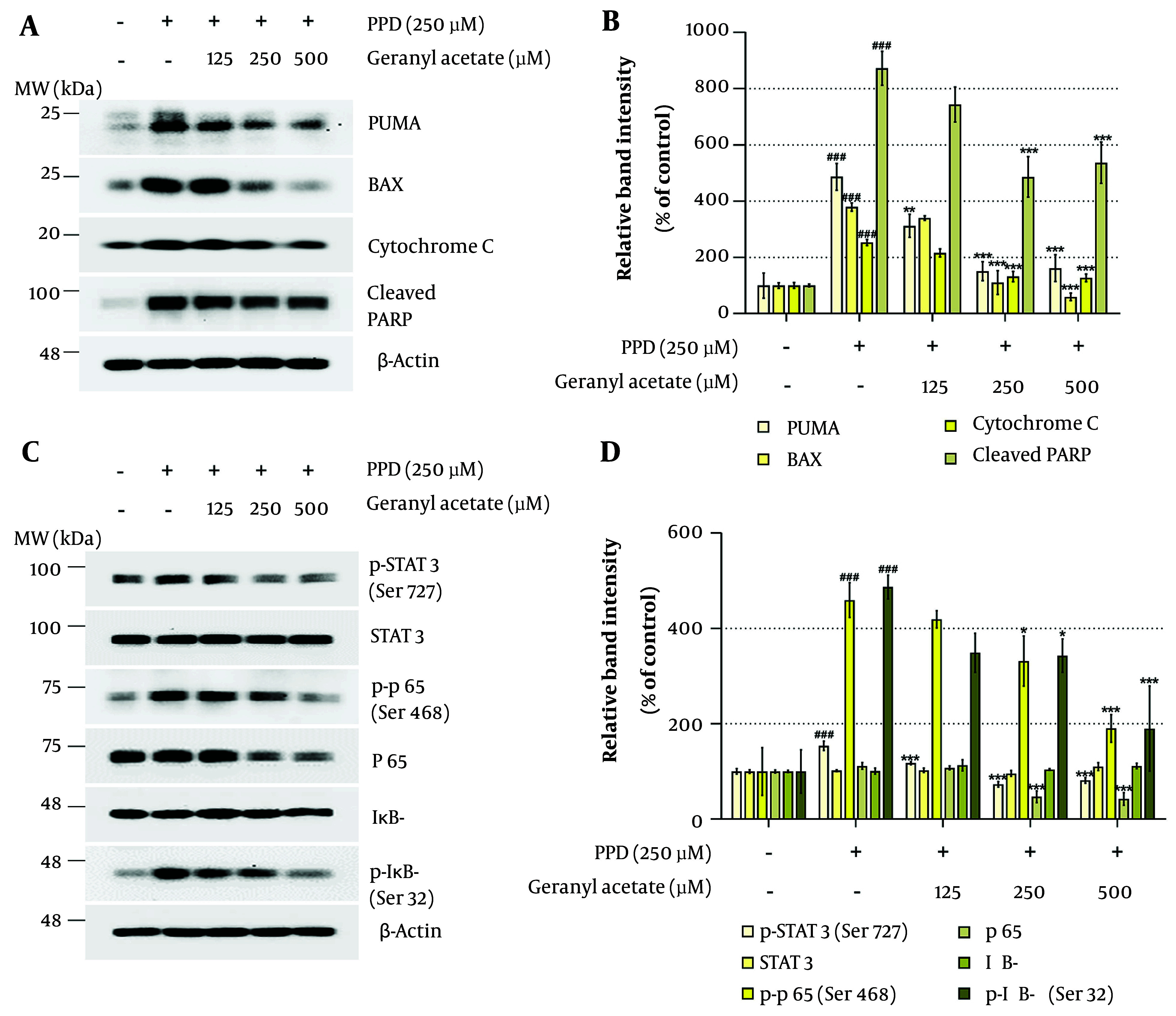
Geranyl acetate (GA) improves para-phenylenediamine (PPD)-induced apoptosis and inflammation signaling in HaCaT cells: A and B, HaCaT cells were seeded in 100 mm dishes (2 × 10^5^ cells/dish) and incubated for 24 hours, followed by treatment with GA (0 - 500 μM), in the presence or absence of PPD (250 μM) for 48 hours. Protein expression levels of the (A) apoptosis-related proteins [BAX, p53 upregulated modulator of apoptosis (PUMA), cytochrome c, and cleaved PARP] and (B) inflammation-related proteins [signal transducer and activator of transcription 3 (STAT3), p-STAT3, p65, p-p65, NF-kappa-B inhibitor alpha (IκB-α), and p-IκB-α] were analyzed by Western blotting. The β-Actin was used as a loading control. Protein band intensities were quantified using ImageJ software (version 1.53t). Data are presented as mean ± standard deviation (SD, n = 3). Statistical significance was determined by one-way analysis of variance (ANOVA) followed by Tukey’s post-hoc test (### P < 0.001 compared with the solvent-treated vehicle control group. * P < 0.05, ** P < 0.01, and *** P < 0.001 compared with the PPD-treated negative control group).

Given that GA was found to suppress DDR signaling (Figure 3), we next evaluated whether it could also attenuate the downstream apoptotic response. Figure 4A and B show the protein expression of key pro-apoptotic markers. Previous studies have demonstrated that the p53 upregulated modulator of apoptosis (PUMA)-BAX-cytochrome c axis mediates mitochondrial apoptosis via caspase-3 activation (48). The PUMA, a BH3-only protein upregulated by p53, binds to and activates BAX, which permeabilizes the mitochondrial outer membrane, leading to cytochrome c release, caspase activation, and PARP cleavage (49, 50).

As shown in Figure 4A and B, GA co-treatment dose-dependently suppressed the PPD-induced upregulation of PUMA, BAX, cytochrome c, and cleaved PARP. These findings support the notion that GA inhibits apoptosis in HaCaT cells subjected to PPD-induced genotoxic stress.

### 4.5. Geranyl Acetate Suppresses Inflammatory Signaling in Para-phenylenediamine-induced HaCaT Cells

**Figure 5.**
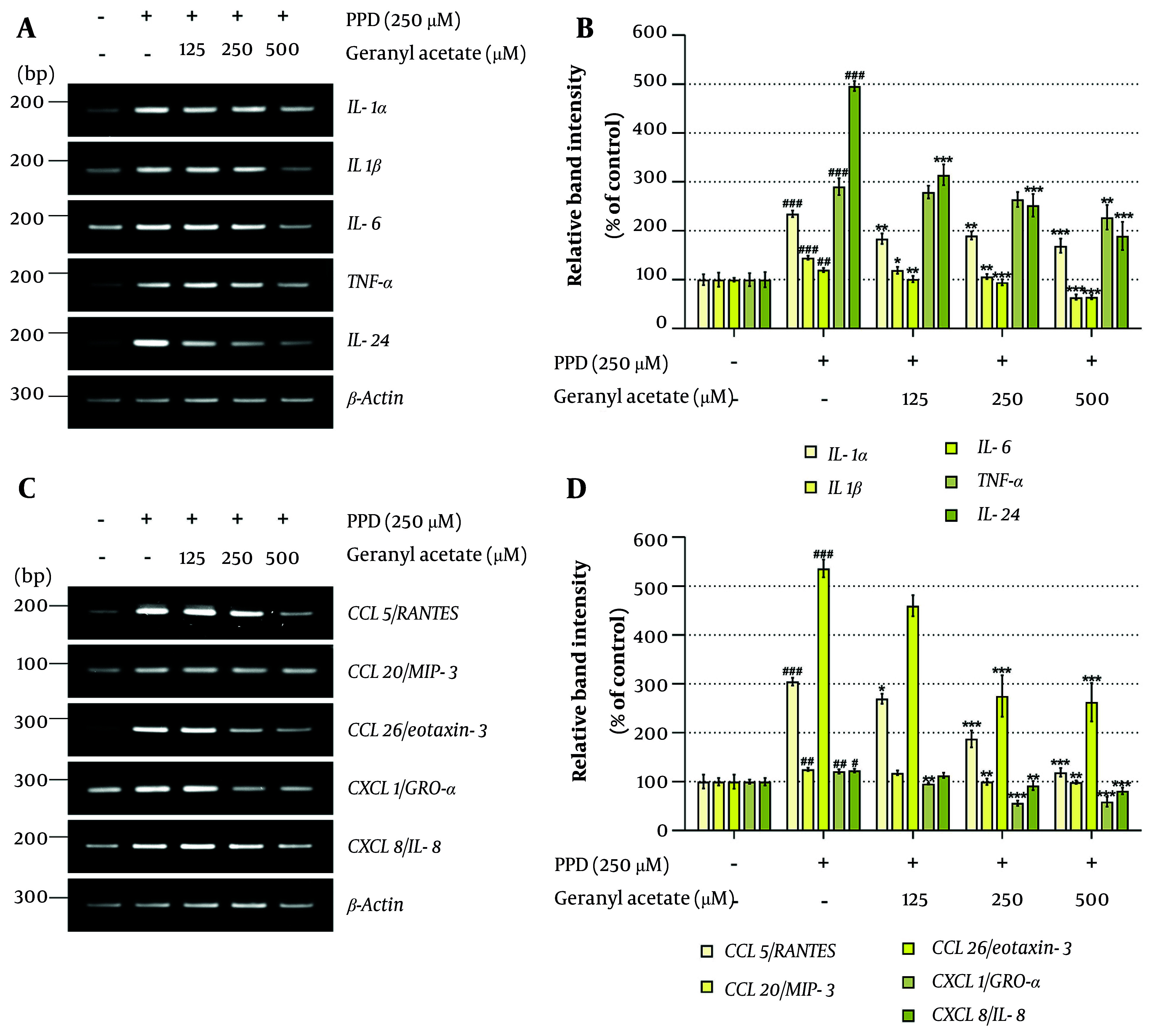
Geranyl acetate (GA) downregulates the mRNA expression level of cytokines and chemokines in para-phenylenediamine (PPD)-stimulated HaCaT cells: A – D, HaCaT cells were seeded in 100 mm dishes (2 × 10^5^ cells/dish) and incubated for 24 hours, followed by treatment with GA (0 - 500 μM), in the presence or absence of PPD (250 μM) for 48 hours. The mRNA expression levels of (A and B) cytokines [interleukin (IL)-1α, IL-1β, IL-6, tumor necrosis factor (TNF)-α, and IL-24] and (C and D) chemokines [C-C motif chemokine ligand (CCL) 5, CCL20, CCL26, C-X-C motif chemokine ligand (CXCL) 1, and CXCL8] were analyzed using reverse transcriptase-polymerase chain reaction (RT-PCR). The β-Actin was used as a loading control. The mRNA band intensities were quantified using ImageJ software (version 1.53t). Data are presented as mean ± standard deviation (SD, n = 3). Statistical significance was determined by one-way analysis of variance (ANOVA) followed by Tukey’s post-hoc test (# P < 0.05, ## P < 0.01, and ### P < 0.001 compared with the solvent-treated vehicle control group. * P < 0.05, ** P < 0.01, and *** P < 0.001 compared with the PPD-treated negative control group).

The DDR-driven MAPK activation has been shown to stimulate the transcriptional activity of signal transducer and activator of transcription 3 (STAT3) and NF-κB p65, key regulators of inflammatory gene expression (51). Given that keratinocyte-mediated inflammation is a crucial contributor to the pathogenesis of inflammatory skin disorders, we examined whether GA could suppress PPD-induced inflammatory signaling. As shown in Figure 4C and D, GA did not alter total STAT3 protein levels but significantly reduced Ser727 phosphorylation in a dose-dependent manner. Similarly, phosphorylation of NF-kappa-B inhibitor alpha (IκB-α, Ser32) and p65 (Ser468), both elevated by PPD, were attenuated by GA. These results suggest that GA inhibits the activation of STAT3 and NF-κB pathways, which are known to drive the expression of pro-inflammatory cytokines and chemokines (52). To further assess the anti-inflammatory potential of GA, we examined mRNA expression of inflammation-related genes. As shown in Figure 5, GA co-treatment dose-dependently suppressed the PPD-induced expression of five pro-inflammatory cytokines [interleukin (IL)-1α, IL-1β, IL-6, tumor necrosis factor (TNF)-α, IL-24] and five chemokines [C-C motif chemokine ligand (CCL) 5/RANTES, CCL20/MIP-3, CCL26/eotaxin-3, (C-X-C motif chemokine ligand) CXCL 1/GRO-α, CXCL8/IL-8]. These findings indicate that GA exerts potent anti-inflammatory effects in PPD-exposed HaCaT cells.

## 5. Discussion

Driven by the growing consumer demand for cosmetic products that cover gray hair or alter hair color, the global oxidative hair dye market continues to expand and is currently valued at approximately $22.5 billion, with a compound annual growth rate (CAGR) of 8.3% (43). Among oxidative hair dye ingredients, PPD is used in more than 80% of products. Despite its well-documented adverse effects, the combination of high efficacy and low cost has made PPD difficult to replace (14). The PPD (molecular weight: 108.1) is rapidly oxidized before skin application, yielding reactive metabolites such as BQDI and BB (53, 54). Upon cutaneous exposure, PPD and its oxidized derivatives readily penetrate the epidermis and reach the basal layer (53, 54). These metabolites can covalently bind to nucleophilic skin proteins, forming hapten-protein complexes that trigger immune responses. Additionally, they may bind to peptides — leading to enzyme depletion — or interact directly with DNA, causing fragmentation and adduct formation (43-45, 53). These mechanisms underlie the association of PPD with carcinogenicity, dermatitis, ACD, and skin barrier dysfunction. Consequently, identifying natural product-derived compounds capable of mitigating PPD-induced cytotoxicity, DDR, apoptosis, and inflammation is of considerable interest (15-19).

In this study, we established a keratinocyte-based in vitro model to assess PPD-induced toxicity. While dendritic cells and T-cells are considered central to ACD pathogenesis, epidermal keratinocytes also contribute significantly by releasing pro-inflammatory cytokines and chemokines that promote immune cell activation, maturation, and migration (55). Moreover, since PPD has been implicated in various cutaneous disorders beyond ACD (56, 57), our keratinocyte-based model provides a relevant platform for evaluating its broader biological effects. Among the 14 plant-derived metabolites tested, GA emerged as a promising candidate due to its protective effects against PPD-induced toxicity in HaCaT cells. Specifically, GA significantly attenuated DDR signaling in a dose-dependent manner by reducing phosphorylation of ATR, p53, and MAPKs. Previous studies have highlighted the therapeutic relevance of inhibiting these pathways in skin cancer prevention and treatment — for example, caffeine may suppress UV-induced skin tumors by inhibiting ATR, while RAF inhibitors such as vemurafenib and sorafenib are effective in treating melanoma (58). Given the reported carcinogenic potential of PPD in various tissues, including the skin (19), the observed anti-DDR effects of GA suggest its potential to mitigate PPD-related tumorigenic risks.

Activation of the DDR pathway leads to phosphorylation of p53, which promotes apoptosis via the PUMA/BAX/cytochrome c cascade and mitochondrial membrane permeabilization. In parallel, MAPK phosphorylation can drive inflammatory signaling through NF-κB and STAT3 (49, 51). In our study, GA significantly downregulated PPD-induced expression of pro-apoptotic markers (PUMA, BAX, cytochrome c, and cleaved PARP), improved cell viability, and reduced LDH release — indicating decreased cell death. In both atopic dermatitis and ACD, T-cell-mediated keratinocyte apoptosis is a major pathogenic feature (59). Notably, keratinocyte apoptosis is a hallmark of eczematous and spongiotic lesions in atopic dermatitis (60). Therefore, the anti-apoptotic effects of GA observed here may hold therapeutic relevance for mitigating keratinocyte damage in a range of PPD-induced skin disorders.

Furthermore, GA co-treatment significantly suppressed phosphorylation of STAT3 and p65 — key regulators of PPD-induced inflammatory signaling — and markedly decreased mRNA expression of several pro-inflammatory cytokines and chemokines. Of particular interest, IL-24, a known biomarker of PPD-induced ACD, was significantly reduced. Previous studies have reported elevated IL-24 expression in PPD-allergic individuals, while IL-24 knockout mice exhibit markedly reduced hypersensitivity responses to PPD (61). Since keratinocyte-derived cytokines and chemokines exacerbate skin inflammation by promoting immune cell infiltration and activation, these molecules represent key therapeutic targets in ACD and related inflammatory conditions (62). Consistent with this, GA significantly downregulated five pro-inflammatory cytokines (IL-1α, IL-1β, IL-6, TNF-α, and IL-24) and five chemokines (CCL5/RANTES, CCL20/MIP-3α, CCL26/eotaxin-3, CXCL1/GRO-α, and CXCL8/IL-8), supporting its potential to alleviate inflammatory responses associated with ACD, atopic dermatitis, eczema, and other PPD-exacerbated skin disorders (18, 63).

Previous studies indicate that oxidative hair dyes typically contain PPD at 2 - 3% (≈ 180 - 280 mM), with EU regulations allowing up to 6% (≈ 550 mM) (19). Given the short application time of hair dyes (≈ 30 minutes), the low concentration (250 μM) and prolonged exposure (≥ 24 hours) used here may not fully reflect real-world conditions (64). However, Nohynek et al. reported that approximately 0.88% of applied PPD can remain on the skin, leading to systemic exposure (65). Hairdressers — who may be exposed up to six times daily and of whom only about one-third use protective gloves — are likely to retain PPD on the skin or in the body for extended periods (66, 67). Because PPD’s toxic and sensitizing effects are well established (19, 68), prior in vitro studies have optimized conditions to reliably reproduce these effects, often using low micromolar concentrations for ≥ 24 hours (56, 57, 69, 70). Following this approach, we applied 250 μM PPD for > 24 hours to ensure reproducibility. Future work should explore higher concentrations with shorter exposures (e.g., 1 hour) in order to better mimic consumer use.

Given our finding that GA attenuates PPD-induced cytotoxicity and inflammation, clinical studies should assess whether formulations containing this compound can protect against PPD-related irritation when combined with hair dyes. As IL-24 is a biomarker not only for contact dermatitis but also for asthma and urticaria (61), its reduction by GA warrants investigation in other relevant cell models, such as bronchial epithelial cells.

### 5.1. Conclusions

In conclusion, this study identifies GA — a naturally derived plant metabolite — as a potent modulator of cytotoxicity, DNA damage, apoptosis, and inflammation in a PPD-stimulated HaCaT keratinocyte model. While these findings provide compelling in vitro evidence of its pharmacological efficacy, further in vivo and clinical investigations are warranted to fully assess the therapeutic potential of GA for managing PPD-induced skin toxicity.

ijpr-24-1-164379-s001.pdf

## Data Availability

The dataset presented in the study is available on request from the corresponding author during submission or after publication.
