# Supplementary figures and images for: Geranyl Acetate Attenuates Para-phenylenediamine-induced Cytotoxicity, DNA Damage, Apoptosis, and Inflammation in HaCaT Keratinocytes
Source: Iran J Pharm Res. 2025 Oct 1;24(1):e164379. doi: 10.5812/ijpr-164379 (PMC12524059; doi:10.5812/ijpr-164379)

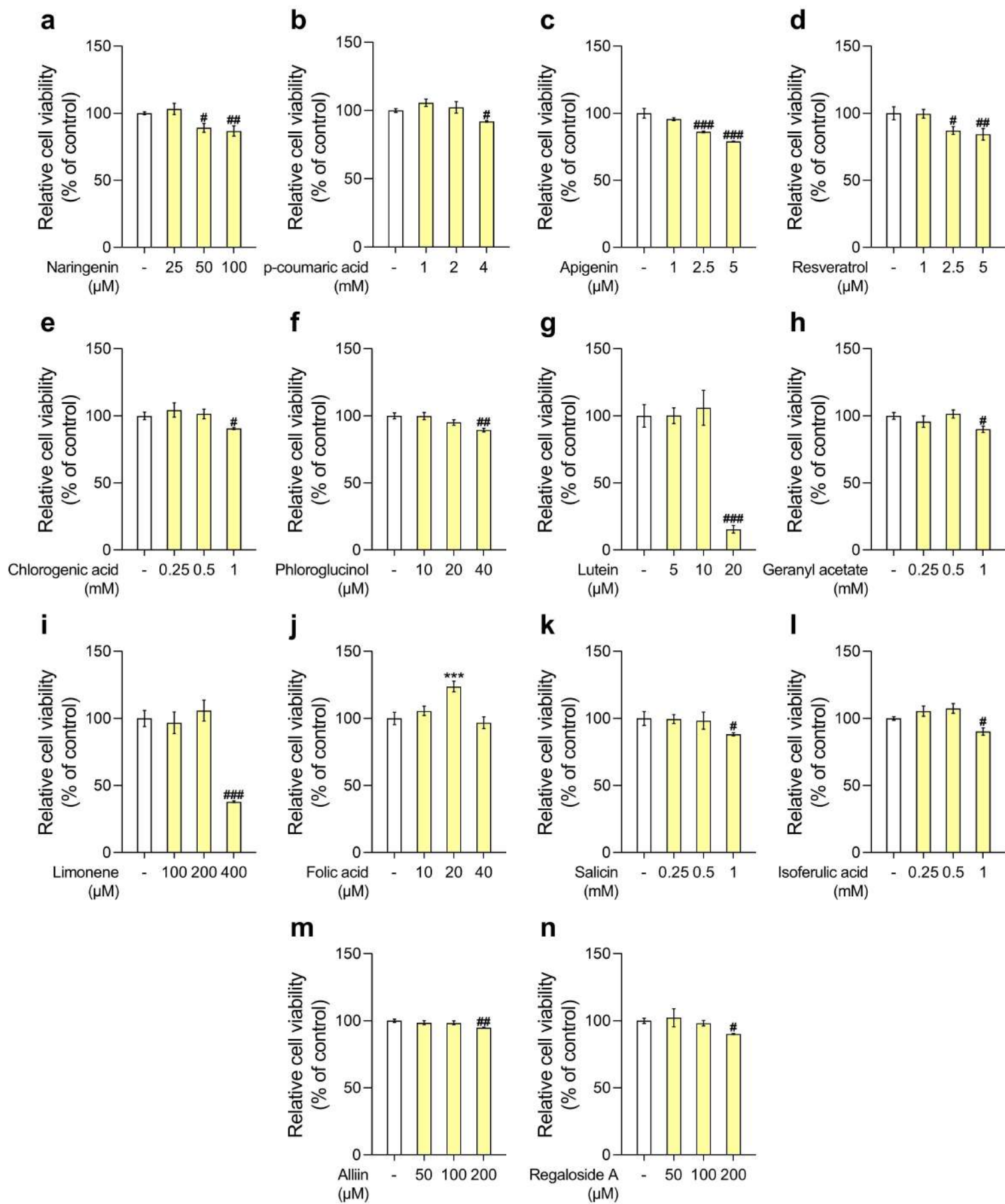

Supplement: ijpr-24-1-164379-s001.pdf [file ijpr-24-1-164379-s001.pdf]
